# Familial CARD14-associated papulosquamous eruption with a novel mutation successfully treated with secukinumab

**DOI:** 10.1016/j.jdcr.2026.01.047

**Published:** 2026-02-03

**Authors:** Jieru Ren, Guozhen Tan, Zhenrui Shi

**Affiliations:** Department of Dermatology, Sun Yat-sen Memorial Hospital, Sun Yat-sen University, Guangzhou, China

**Keywords:** CARD14-associated papulosquamous eruption, novel mutation (p.Met119Lys), pityriasis rubra pilaris, secukinumab

## Introduction

CARD14-associated papulosquamous eruption (CAPE) is an uncommon autosomal-dominant inflammatory dermatosis. It typically presents early in life with diffuse, psoriasis-like eruptions, palmoplantar keratoderma, and a difficult-to-treat course that often responds poorly to conventional therapies. CAPE is driven by gain-of-function variants in CARD14, which promote persistent NF-κB signaling and amplify interleukin-17–skewed inflammatory pathways.^1^ Multiple pathogenic CARD14 variants have been reported in association with CAPE, underscoring substantial phenotypic variability and ongoing therapeutic limitations.^2^ In this report, we describe an infrequent familial presentation involving a father and daughter with severe disease who carried a previously unreported CARD14 variant (c.356T>A; p.Met119Lys). Both individuals demonstrated marked clinical improvement after receiving secukinumab, a monoclonal antibody targeting IL-17A.

## Case report

**Fig 1:**
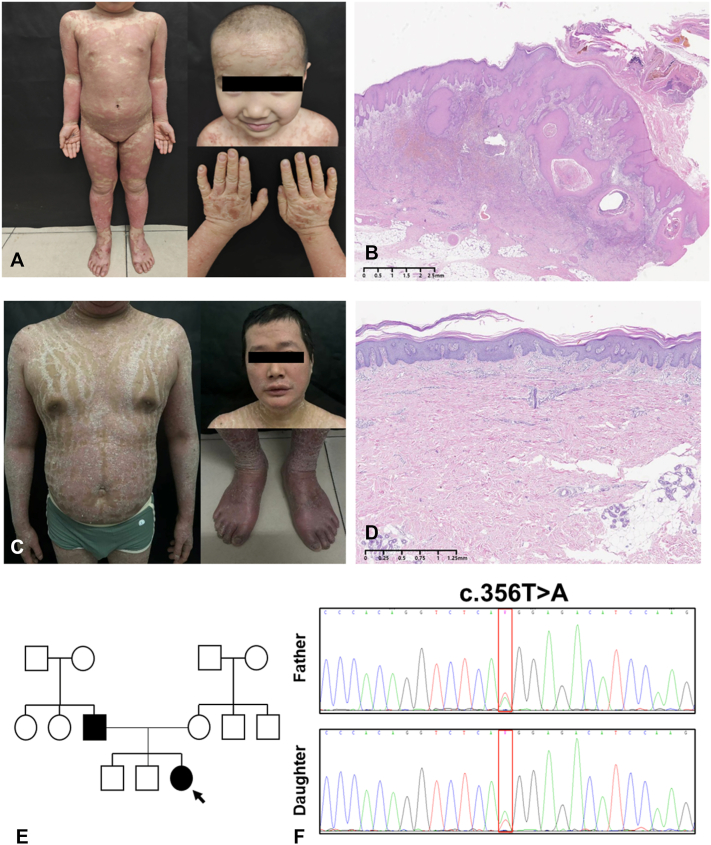
**A,** Skin manifestations of the daughter and **(B)** histologic features of biopsy specimens from buttock. **C,** Skin manifestations of the father and **(D)** histologic features of biopsy specimens from abdomen. **E,** Pedigree chart of the family, with the proband indicated by an *arrow*. **F,** Sanger sequencing chromatograms showing the heterozygous CARD14 variant (c.356T>A, p.Met119Lys) in both the daughter and her father.

A 4-year-old girl presented with a 3-year history of pruritic, erythematous, scaly eruptions involving the face and extremities, with a notable flare over the prior 2 weeks. The eruption began at 9 months of age as facial erythematous patches, intermittently accompanied by serous oozing. Over time, lesions spread to the extensor aspects of the knees and the buttocks, with progressive pruritus. Persistent excoriation led to lichenification, along with coarse hyperkeratosis of the palms and soles. The disease course was seasonal, with worsening during winter months. Prior therapies, including oral antihistamines, topical sulfur ointment, and emollients, provided only transient symptomatic benefit without sustained disease control. Physical examination revealed diffuse erythematous, scaly plaques (Fig 1, *A*). Skin biopsy showed psoriasiform epidermal hyperplasia with parakeratosis and superficial perivascular lymphocytic inflammation (Fig 1, *B*).

The patient’s father, a 33-year-old male, had generalized erythematous and scaly lesions since infancy, persisting for over 30 years (Fig 1, *C*). Lesions predominantly involved the trunk, limbs, face, and scalp, with intervening unaffected skin, and were largely nonpruritic. Progressive bilateral ectropion developed over time. Seasonal variation was noted, with winter exacerbations marked by thickened scales, painful fissures, and occasional bleeding, and summer flares characterized by increased flushing. Diagnosed with pityriasis rubra pilaris at our clinic, he had previously declined systemic therapy due to financial constraints. Intermittent topical erythromycin provided only mild relief. Skin biopsy revealed acanthosis, focal parakeratosis, and mild spongiosis (Fig 1, *D*).

**Fig 2:**
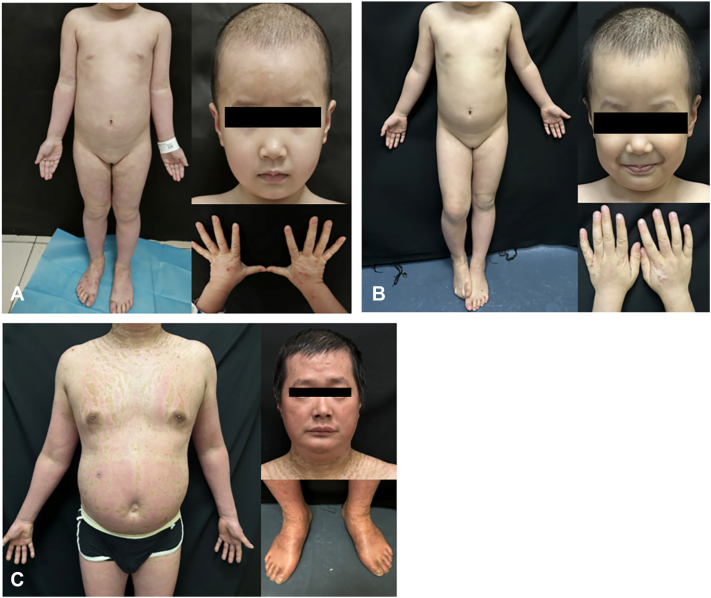
Clinical images of the daughter at **(A)** 1 week and **(B)** 4 weeks after a single subcutaneous dose of secukinumab. **C,** Clinical image of the father at 4 weeks after a single subcutaneous dose of secukinumab.

No other relatives were affected (Fig 1, *E*). Whole-exome sequencing identified a heterozygous CARD14 mutation (c.356T>A, p.Met119Lys) in both the daughter and father (Fig 1, *F*), confirming the diagnosis of CAPE. The daughter initiated subcutaneous secukinumab (150 mg monthly) after written informed consent and achieved complete resolution of scaling, pruritus, and erythema within 4 weeks (Fig 2, *A* and *B*). After genetic confirmation and observing her rapid clinical response, the father agreed to start secukinumab (300 mg monthly) and experienced similar improvement within 4 weeks (Fig 2, *C*).

## Discussion

To our knowledge, this is a rare familial presentation of CAPE associated with a novel CARD14 variant, p.Met119Lys. The marked clinical improvement observed with secukinumab therapy in these patients underscores the critical role of IL-17A in the inflammatory cascade of CAPE.^3^ Although previous reports have documented other CARD14 mutations, such as c.349+2T>C, responding favorably to IL-17 inhibitors,^4^^,^^5^ familial cases with the specific p.Met119Lys variant have not been previously described.

Clinically, CAPE typically presents with early onset, significant facial involvement, and a variable range of lesions including psoriasiform plaques, follicular papules, and palmoplantar keratoderma. These clinical features help distinguish CAPE from classic psoriasis and pityriasis rubra pilaris, whereas histopathologic findings may overlap among these entities.^6^

CAPE is often refractory to standard approaches, including topical corticosteroids, systemic retinoids, and traditional immunosuppressants, with many patients experiencing incomplete or short-lived benefit. In contrast, accumulating evidence supports improved disease control with biologics that inhibit the IL-23/IL-17 pathway, such as secukinumab, ixekizumab, ustekinumab, and guselkumab.^2^ For example, secukinumab has been associated with rapid and pronounced clinical responses in both pediatric and adult CAPE cases, with some patients reaching PASI 90 within a timeframe of weeks to a few months.^4^ Similarly, ustekinumab^1^^,^^7^ and guselkumab^8^ have demonstrated clinical efficacy, though some patients required increased dosages or shorter dosing intervals to maintain therapeutic response. Ixekizumab also provided significant symptom resolution in reported cases.^5^^,^^9^ Taken together, available evidence suggests that biologic agents—particularly those targeting the IL-23/IL-17 pathway—represent a promising, individualized therapeutic avenue for CAPE, underscoring the need to align treatment selection with patient-specific clinical and molecular features. Emerging mechanistic data are consistent with this strategy. CAPE lesions have been reported to exhibit increased local IL-17A expression, whereas IL-23A may be elevated in the circulation of a subset of patients, a pattern that could be associated with heightened sensitivity to IL-23–directed therapy. In addition, altered expression of epidermal differentiation markers, including involucrin and TGM1, has been described, supporting the concept of disordered keratinocyte maturation downstream of CARD14-driven signaling.^10^ These molecular features may serve as future biomarkers to predict therapeutic response and facilitate personalized treatment decisions. Further research is warranted to clarify their predictive value.

### Declaration of generative AI and AI-assisted technologies in the writing process

During the preparation of this work, we used ChatGPT in order to improve readability and language of the manuscript. After using this tool, the authors reviewed and edited the content as needed and take full responsibility for the content of the published article.

## Conflicts of interest

None disclosed.
